# A unifying model for membrane protein biogenesis

**DOI:** 10.1038/s41594-024-01296-5

**Published:** 2024-05-29

**Authors:** Ramanujan S. Hegde, Robert J. Keenan

**Affiliations:** 1 Cell Biology Division, MRC Laboratory of Molecular Biology, Cambridge, UK; 2 Gordon Center for Integrative Science, The University of Chicago, Chicago, IL, USA

## Abstract

Alpha-helical integral membrane proteins comprise ~25% of the proteome in all organisms. The membrane proteome is highly diverse, varying in the number, topology, spacing and properties of their transmembrane domains. This diversity imposes different constraints on the insertion of different regions of a membrane protein into the lipid bilayer. Here, we present a cohesive framework to explain membrane protein biogenesis wherein different parts of a nascent substrate are triaged between Oxa1 and SecY family members for insertion. In this model, Oxa1 family proteins insert transmembrane domains flanked by short translocated segments whereas the SecY channel is required for insertion of transmembrane domains flanked by long translocated segments. Our unifying model rationalises evolutionary, genetic, biochemical and structural data across organisms and provides a foundation for future mechanistic studies of membrane protein biogenesis.

## Introduction

The transfer of molecules and information between the inside and outside of a cell relies on integral membrane proteins of diverse topology and characteristics (Box 1). The core processes of membrane insertion would have existed at the plasma membrane of the last universal common ancestor^1^. The plasma membrane remains the site of membrane protein insertion in bacteria and archaea. The endoplasmic reticulum (ER), which likely evolved from the archaeal plasma membrane^2^, is the major site of membrane protein insertion in eukaryotes. Mitochondria and plastids evolved from bacteria^3^, so their inner membranes descended from the ancestral bacterial plasma membrane. All of these evolutionarily related membranes have members of the Oxa1 family^4,5^ or SecY family^6^ (Box 2), the only known membrane protein insertion factors that trace back to the last universal common ancestor^7^.

The evolutionary path of membrane protein insertion presumably progressed from an unassisted insertion reaction to a process facilitated by insertion factors. This transition broadened the range of proteins that could be inserted into membranes, which in turn allowed the evolution of more elaborate and diversified insertion machinery. By considering how membrane insertion and its accompanying machinery arose, we arrive at a unifying model for membrane protein biogenesis that accommodates the current diversity of the membrane proteome across all organisms. In this model, Oxa1 facilitates insertion of transmembrane domains (TMDs) flanked by a short translocated domain, whereas SecY is required for insertion of TMDs followed by a long translocated domain. Proteins of diverse topology and properties can be accommodated by dynamically toggling between SecY and different Oxa1 family members during their co-translational insertion.

### The evolution of membrane insertion

**Fig. 1:**
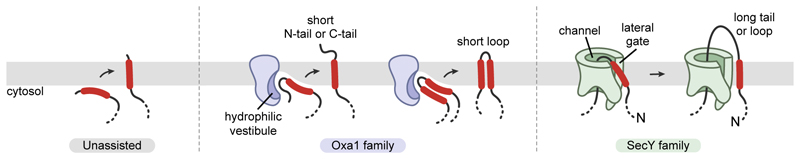
Mechanisms for alpha-helical membrane protein insertion. (Left) Unassisted membrane protein insertion can occur if the energetically favoured reaction of transmembrane domain (TMD, red) partitioning into the hydrophobic membrane offsets the penalty for translocation of a short flanking segment of hydrophilic polypeptide. (Middle) Oxa1 family members use a hydrophilic vestibule to facilitate translocation of short tails and loops during insertion of one or two TMDs. (Right) SecY family members use a central channel to initiate the translocation of long tails and loops concomitant with TMD insertion via a lateral gate.

Theoretical and experimental studies show that the energetically favourable reaction of partitioning a hydrophobic transmembrane domain (TMD) into the lipid bilayer is sufficient to offset the penalty of translocating a short segment of flanking hydrophilic polypeptide across the membrane^8–11^. This can be achieved for a single TMD with a short ‘tail’ at the N- or C-terminus (Fig. 1, left), or two TMDs with a short intervening loop. This would have been the ancestral mechanism of membrane protein insertion. The substrate range of ‘unassisted’ insertion would presumably have been very limited, with insertion being strongly competed by insolubility and aggregation, especially for multi-TMD proteins.

Expansion of the substrate range for the unassisted mechanism could have occurred by evolving a ribosome receptor that allows synthesis close to the membrane. Such a receptor could simply have been a ribosome-binding peripheral or single-TMD protein that engages near the polypeptide exit tunnel. Membrane-proximal protein synthesis would have facilitated co-translational insertion of multi-TMD proteins by successively inserting TMD pairs as they emerge from the ribosome. Each newly emerging TMD would rapidly bind to the adjacent membrane surface^12^, reducing its exposure to the bulk cytosol. By having only two membrane-associated TMDs exposed at any time, the possibility of substrate aggregation into translocation-incompetent states is reduced.

Although the substrate range would still be limited to short translocated tails and loops, this mechanism is compatible with either topology: the first TMD could insert by itself concomitant with N-tail translocation, or insert as a pair with the next TMD concomitant with translocation of the intervening loop. Experiments with liposomes or nanodiscs show that various multipass membrane proteins can indeed be inserted without any insertion factors, albeit with low efficiency due to competing aggregation^13–16^. Not surprisingly, the insertion must occur co-translationally^17^, is favoured by high membrane concentration^18^, and is only compatible with substrates containing short translocated loops and tails.

A membrane protein that reduces the energetic barrier for hydrophilic segment translocation would relax the constraint on translocated domain length, allowing for translocation of longer tails and loops (Fig. 1, middle). The Oxa1 family is thought to achieve this by providing a hydrophilic vestibule that penetrates part of the way into the membrane^4,19,20^. The vestibule locally thins the membrane^21^, reducing the barrier to translocation of a hydrophilic segment from this site. Importantly, the universally conserved core Oxa1 fold, a simple three-TMD bundle with short translocated hydrophilic segments^5,7,20,22^, could have evolved at a time when only the ancestral insertion mechanism was possible.

Once an Oxa1-like protein had evolved, the substrate range could be expanded in two ways. First, a lower barrier to flanking domain translocation would allow insertion of lower hydrophobicity TMDs. Second, flanking domains could be longer. These relaxed features would allow the evolution of increasingly complex and diverse membrane proteins. Oxa1 members have been shown to translocate hydrophilic segments of up to ~50 amino acids, depending on composition and folding propensity of the translocated domain, hydrophobicity of the flanking TMD(s), and features of the specific Oxa1 family member^4,20^. Diversification of the Oxa1 family has allowed some members to accommodate longer translocated domains for certain substrates^23^.

### From membrane insertion to secretion

Translocation of hydrophilic segments longer than ~100 amino acids must generally use a membrane-spanning channel. This role is served by the SecY family, a pseudosymmetric protein whose two homologous halves house a channel between them^24^. Structural and sequence analysis suggests that the core of each SecY half arose from an Oxa1-like ancestor^7^. Duplication, fusion, and anti-parallel interaction of this ancestor would have juxtaposed two hydrophilic vestibules that evolved into a transmembrane channel capable of translocating long hydrophilic domains across the membrane.

Like the Oxa1 family from which it may have evolved, hydrophilic domain translocation by SecY is coupled to membrane insertion of an adjacent hydrophobic domain. Rather than the hydrophilic segment passing through a locally distorted membrane, it is pulled into SecY’s membrane-spanning aqueous channel^25,26^. The hydrophobic domain achieves this by accessing the membrane via a lateral gate in SecY such that its downstream flanking domain enters the central channel in a looped configuration (Fig. 1, right). Because hydrophobic domain binding at the lateral gate is coupled to channel opening, the initiation of translocation is coupled to membrane insertion.

Once the SecY channel has been opened and the initial downstream segment of hydrophilic polypeptide has been threaded through, there is no limit to the length of protein that can move across the membrane^24^. Translocation ends and the channel reverts to its inactive closed state in one of two ways: termination of translation or emergence of a downstream TMD. Each of these is described in turn.

Translation termination allows translocation of the polypeptide’s C-terminus through SecY. This C-terminal translocated domain would remain anchored to the membrane by the preceding hydrophobic domain. The evolution of a membrane-bound protease that liberates the membrane-embedded hydrophobic domain would have led to the invention of protein secretion. The enzyme that carries out this reaction, called signal peptidase, is specific for particularly short hydrophobic domains known as signal peptides^27,28^.

Emergence of a downstream TMD from the ribosome allows the TMD to enter SecY and pass through its lateral gate into the membrane^29,30^. In this way, a long loop of polypeptide between the translocation-initiating hydrophobic domain and translocation-terminating TMD is translocated to the non-cytosolic side of the membrane. If the translocation-initiating hydrophobic domain is a signal peptide, it is proteolytically cleaved to liberate the new N-terminus on the trans site of the membrane. Thus, a long segment of hydrophilic polypeptide can be translocated through SecY as long as it is preceded by a hydrophobic domain that engages SecY’s lateral gate.

### A general model for membrane protein biogenesis

With mechanisms for translocating short hydrophilic tails and loops via Oxa1 and long hydrophilic domains through SecY, one can rationalise how the two together can mediate insertion of the full topologic range of membrane proteins (Box 1). To better convey the overall concept, we describe this framework using the general terms Oxa1 and SecY, rather than species-specific nomenclature (which is summarized in Box 2). The initial step is targeting of the nascent membrane protein to the lipid bilayer^31^. This typically occurs co-translationally and is mediated by the first hydrophobic segment, either a signal peptide or TMD. This element is engaged by the signal recognition particle (SRP) and delivered to a receptor at the membrane, where the remainder of the protein is synthesized. When the only hydrophobic element(s) are within ~70 amino acids of the C-terminus, targeting occurs post-translationally, aided by cytosolic factors that keep the substrate soluble until its arrival at the membrane^32–36^. After targeting, the TMD(s) are inserted concomitant with flanking domain translocation.

Membrane proteins without any long translocated domains can be inserted by Oxa1 family member(s) and do not require SecY’s lateral gate or central channel^37–42^. This typically occurs co-translationally for all TMDs except those near the C-terminus, which are inserted post-translationally^34,39,43^. Because TMDs flanked by short translocated domains can also insert unassisted^44,45^, albeit with lower efficiency and increased risk of aggregation, the Oxa1 requirement for many substrates is not absolute. Proteins with one or more long translocated domains require SecY, with the preceding hydrophobic domain initiating translocation by engaging SecY’s lateral gate^46^.

**Fig. 2:**
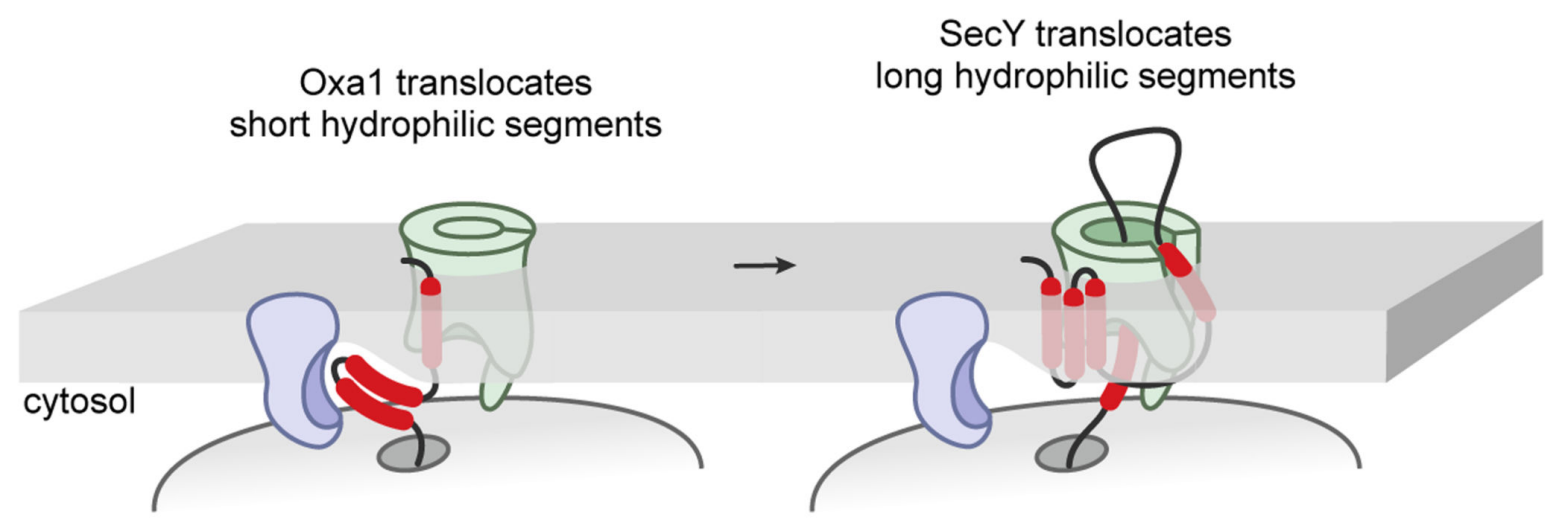
Oxa1 and SecY collaborate during multipass protein translocation. The ribosome serves as a binding platform for Oxa1 and SecY modules during multipass protein biogenesis. A nascent chain emerging from the ribosome can toggle between Oxa1 for translocation of short hydrophilic segments and SecY for translocation of long hydrophilic segments.

Membrane proteins with multiple TMDs and translocated domains of different lengths use both Oxa1 and SecY for different regions as dictated by translocated domain length. TMDs close to the N- or C-terminus with a short translocated tail use an Oxa1 family member operating co- or post-translationally, respectively^40,43^. All other TMDs are inserted co-translationally by a membrane-bound ribosome using Oxa1 for short translocated loops and SecY for long translocated loops and termini^46,47^. During the co-translational phase of biogenesis, the ribosome provides a binding platform for both Oxa1 and SecY family members^46,48^, allowing the nascent chain to access the appropriate factor suited for each segment of polypeptide^46^ (Fig. 2).

**Fig. 3:**
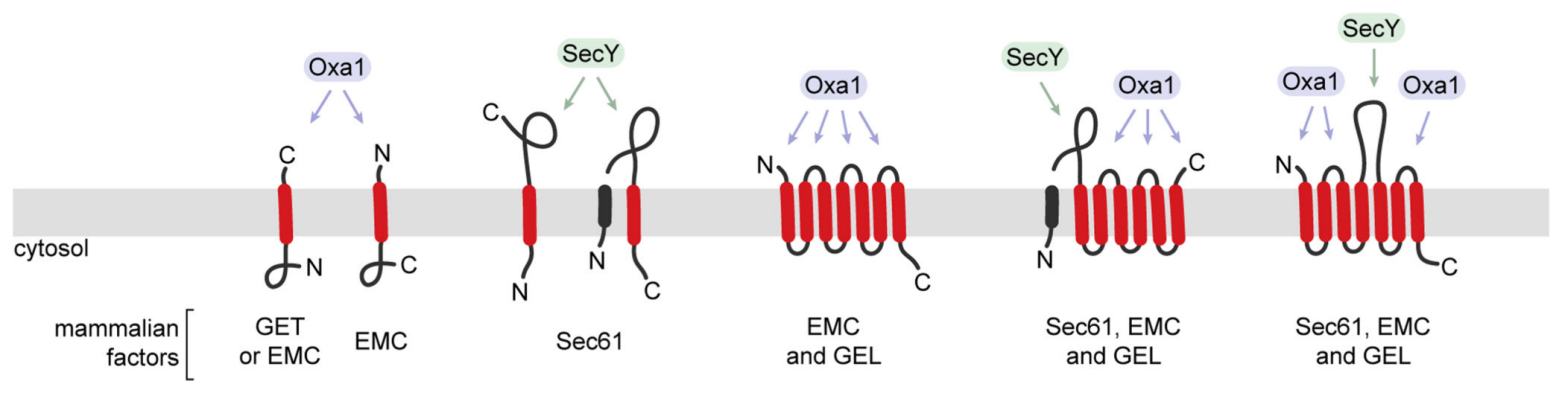
Division of labor between Oxa1 and SecY accommodates a diverse membrane proteome. SecY and Oxa1 family members work together on different parts of many multipass proteins, but can also work separately on other types of proteins. The translocated tails and loops of topologically diverse membrane proteins are labelled by which type of protein (Oxa1 or SecY family) would mediate their translocation. The specific factors thought to be responsible for the different types of translocation in the mammalian ER are indicated below (GET, EMC, and GEL are Oxa1 family members and Sec61 is a SecY family member).

What emerges is a unified model where biogenesis of a membrane proteome uses Oxa1 and SecY for the translocation of short and long segments of hydrophilic polypeptide, respectively (Fig. 3). Given that Oxa1’s activity can be replaced by an unassisted mechanism (albeit with lower efficiency), especially in the context of a membrane-bound ribosome, its loss can be tolerated for some substrates or in some cells. SecY’s translocation activity cannot be compensated, so it is only dispensable for substrates whose translocated hydrophilic domains are all short. Thus, the SecY family is needed for translocation of long hydrophilic domains, whereas Oxa1 family members carry out the bulk of insertion reactions given that the median length of membrane protein translocated domains is ~20 amino acids in all organisms. This would explain why bacteria, yeast, mammalian cells, and endosymbiont organelles are each severely compromised or inviable when their Oxa1 family member(s) are eliminated^4,41,49,50^. The essentiality of secretion would explain why SecY deletion is inviable across all organisms^24^.

### Experimental support for the unifying model

A wide range of genetic, biochemical, structural and evolutionary data across experimental systems can be rationalized by the unifying model of membrane biogenesis proposed here. The experimental and predicted structures of diverse Oxa1 family members in bacteria, archaea, endosymbiont organelle inner membranes and the ER show a cytosol-facing hydrophilic vestibule that would lower the barrier for translocation of short domains, but no channel that could support translocation of long domains^7,19,20,22,46,48,51–57^. By contrast, structures of prokaryotic and eukaryotic SecY family members show a reversibly plugged translocation channel and an adjacent lateral gate where a hydrophobic signal has been observed in bacterial and mammalian systems^24–26,58^.

Translocation of long hydrophilic domains coupled to membrane insertion of a preceding hydrophobic domain is strictly dependent on SecY based on immunodepletion experiments in vitro^40,59^, SecY inactivation or mutation experiments in cells^60–65^, and sensitivity to inhibitors^46,66–73^ that bind to and occlude the SecY lateral gate^74,75^. Membrane insertion of a few such substrates can be reconstituted with purified SecY in proteoliposomes^59,70,76–81^, a reaction that cannot proceed if the lateral gate is covalently locked by a disulfide bond^82^. Although many of these studies used a cleavable signal peptide as the hydrophobic domain, the extrapolation to a TMD engaging the lateral gate in the same topology is compelling. Thus, SecY is both necessary and sufficient for long domain translocation initiated by lateral-gate-mediated membrane insertion of a preceding hydrophobic domain.

By sharp contrast, SecY lateral gate inhibitors do not impact membrane proteins with short translocated domains^40,43,46,66,67,71–73,83^. Immunodepletion of SecY from mammalian ER microsomes had little or no effect on co-translational insertion of N-terminal TMDs preceded by a short translocated tail^40^ or post-translational insertion of C-terminal TMDs followed by a short translocated tail^34,40,44,84^. Sec-independent post-translational insertion of a C-terminal TMD was also shown in *S. cerevisiae*^85^. The insertion of a number of membrane proteins, each with only short translocated domains, are unaffected upon acute SecY depletion in *E. coli*^41,86–90^. Although other such proteins are impacted by SecY depletion^77,86,91–93^, interpretation of this result warrants caution because the SecY requirement could reflect its ribosome binding function and not that it’s channel or lateral gate were used for insertion^46^. Despite this caveat, TMD insertion coupled to translocation of a short flanking domain generally occurs via route(s) that do not depend on the SecY channel or lateral gate.

Conversely, depletion of Oxa1 family members in bacteria^41,63–65,88–90,92–95^, inner endosymbiont organelle membranes^42,96–100^, and the ER^39,40,43,46,47,101^ impairs biogenesis of membrane proteins containing short translocated domains. This applies to N-tails, internal translocated loops, and C-tails. Extending these elements either precludes translocation or makes their translocation dependent on SecY via the preceding hydrophobic domain^43,46,102–104^. N- and C-tails can be physically crosslinked to the hydrophilic vestibule of both bacterial and eukaryotic Oxa1 family members prior to tail translocation^43,105,106^. SecY-mediated initiation of translocation by a preceding hydrophobic domain and Oxa1-mediated translocation of short domains by adjacent TMD(s) has been reconstituted with purified prokaryotic and eukaryotic family members^37,39,40,59,77,78,80,81,107^.

All membrane systems derived from the plasma membrane of the last universal common ancestor would have originally contained Oxa1 and SecY^7,108^ (Box 2). In the inner mitochondrial membrane where long domains are no longer translocated from the matrix, SecY (but not Oxa1) is almost always lost. By contrast, SecY and Oxa1 are both retained in the inner membranes of plastids where translocation of long domains from the stroma is still required. The selective retention of Oxa1 upon loss of long-domain-translocation implies that SecY cannot effectively fulfil membrane insertion reactions typically carried out by Oxa1. One reason might be that opening a closed SecY seems to be slow in native membranes^109,110^, so the risk of losing translocation competence would be high when multiple TMDs are separated by short intervening segments. By contrast, the simple architecture of Oxa1 would allow insertion to occur sufficiently rapidly to keep up with translation. It is also possible that two closely spaced TMDs would require the second TMD to be pulled into SecY’s hydrophilic channel, an energetically unfavoured reaction that impedes lateral gate engagement by the preceding TMD. By inserting both TMDs together, Oxa1 would bypass this problem.

### The paradigm of the mammalian ER

Division of labour between Oxa1 and SecY for the range of substrates that comprise the membrane proteome has been demonstrated in bacteria, yeast, and mammals. The mechanistic basis of such division, and the cooperation between Oxa1 and SecY during multipass membrane protein biogenesis, is best understood in mammals. The mammalian ER contains three Oxa1 family members as part of larger complexes called GET, EMC and GEL^7,22,46,47,53^. The core of each complex contains an Oxa1 protein (GET1, EMC3, and TMCO1, respectively) associated with an obligate partner (GET2, EMC6, and OPTI, each of which share an evolutionary origin). The Oxa1 members and their partners derive from archaeal ancestors^7,52^. The SecY family members in eukaryotes are known as Sec61^24^.

GET, EMC and GEL collectively mediate insertion of TMDs flanked by short translocated domains, whereas Sec61 mediates insertion of TMDs that are followed by long translocated domains. The choice between GET, EMC and GEL for TMD insertion depends on its context and hydrophobicity. GET is used for tail-anchored (TA) proteins with a high-hydrophobicity TMD^33,34,39,111–114^. This specificity is imposed by GET3, the targeting factor that delivers TA proteins to the GET1-GET2 complex. TA proteins of lower hydrophobicity use EMC for insertion^39^. The overlap between GET and EMC among TA protein substrates is substantial, so only the most and least hydrophobic TMDs are strongly reliant on GET and EMC, respectively. This redundancy, together with at least some capacity for unassisted TA protein insertion^44,45^, explains why neither GET nor EMC is strictly essential at the single cell level but shows strong synthetic fitness costs^49^.

The remaining single-pass membrane proteins target to the membrane co-translationally using SRP, then use either EMC or Sec61 for TMDs flanked by short or long translocated domains, respectively. EMC mediates the co-translational insertion of TMDs preceded by a translocated N-tail of ~50 amino acids or less^40,83,105^, whereas Sec61 mediates insertion of all other non-TA single-pass membrane proteins^59,71,72^. This clear segregation of pathways is supported by experiments using Sec61 lateral gate inhibitors, which only inhibit the latter class of proteins. Triage between these two routes occurs shortly after targeting^105^, when nascent substrates first sample EMC for potential insertion before ribosome docking at Sec61. Thus, all single-pass proteins with a short translocated domain use an Oxa1 family member (either GET or EMC), whereas those with a long-translocated domain use the Sec61 channel. This same segregation likely applies to *S. cerevisiae* (which has both GET and EMC) and *E. coli* (which contains YidC as its sole Oxa1 family member).

The insertion machinery used by multipass membrane proteins is also dictated by the length of its translocated domain(s). For proteins containing both short and long translocated domains, more than one factor is employed during insertion. If the first translocated domain is short, EMC would be employed for insertion of the first (or first two) TMD(s) before ribosome docking at Sec61. Once docked on Sec61, subsequent insertion of pairs of TMDs proceeds by one of two routes. If the loop between them is short, the TMD pair is inserted by the ribosome-associating GEL complex^46,47^, which is part of a larger multipass translocon^20,46–48,115^. If the loop between them is long, the first TMD engages the Sec61 lateral gate, the loop is translocated through the Sec61 channel, and the second TMD inserts via the lateral gate. Although many eukaryotes, such as S. cerevisiae, do not contain the GEL complex, EMC could potentially fulfil this role.

The mechanism of toggling between Sec61 and GEL is not clear but seems to involve the PAT complex, a ribosome-binding chaperone conserved widely across eukaryotes^46,47,116^. Part of the ribosome-binding domain of the PAT complex is positioned between Sec61 and the ribosome to preclude opening of the Sec61 lateral gate^46^. In this configuration, the substrate would be directed for insertion to GEL, which sits adjacent to PAT. Emergence of a long loop downstream of a TMD might displace PAT to allow that TMD to engage Sec61’s lateral gate^46^. The key helix of PAT that blocks Sec61 opening also protrudes into the ribosome exit tunnel, suggesting a potential mechanism by which accumulation of a non-translocated loop of substrate can trigger PAT displacement.

Insertion of pairs of TMDs via either GEL or Sec61 continues until termination. If there remains a final TMD whose C-terminal flanking domain needs to be translocated, the path used again depends on C-tail length^43^. Those longer than ~50 amino acids use Sec61’s lateral gate (as shown by its sensitivity to a Sec61 inhibitor), whereas shorter tails are translocated via EMC by a mechanism similar to TA protein insertion. A multipass protein can therefore toggle back and forth between different Oxa1 family members and Sec61 depending on the TMD’s context. For example, a protein could begin insertion using EMC, then a combination of GEL and Sec61, and come back to EMC for the final TMD. Despite this complexity, the principle remains straightforward: the SecY family is needed when translocated domains are long, and Oxa1 family members are used when translocated domains are short.

We propose that this unifying principle applies across all life and reflects the evolutionary origins and conservation of the Oxa1 and SecY families. Both the Oxa1 family and SecY family are essential at the cell level across organisms. This is easily seen in organisms, such as *E. coli,* where only one of each family member is present, but is only now emerging in the eukaryotic ER where multiple Oxa1 members afford some degree of redundancy and robustness. Although various nuances in this paradigm will undoubtedly emerge, interpretations of past results and future studies will benefit from the guiding principle of a division of function between the Oxa1 and SecY families based on length of the flanking translocated domain.

### Future challenges

The body of evidence marshalled to support our unifying framework for membrane protein insertion derives from many experimental systems employing a range of model substrates. Although such diverse sources of data affords a degree of robustness to our model, it will be important to now test each part of the model in a systematic manner in key prokaryotic and eukaryotic systems. Molecular dissection is best done in well-controlled and precisely manipulable biochemical systems, with proteome-wide analyses being used to generalise the findings. This is analogous to how the core principles and mechanisms of SRP-mediated targeting derived from detailed analysis of model substrates^117^, followed much later by proteome-wide validation^118–120^. The challenge now is to use fully reconstituted systems of membrane insertion to define the activities and limitations of each key factor, then use global *in vivo* analyses to corroborate the findings and reveal gaps in our understanding.

A related challenge is to reconstruct the hypothesised evolutionary path of membrane insertion using a series of increasingly complex membrane protein insertion factors. This would begin with an empty liposome system capable of simple membrane insertion at low efficiency^44,45^ and progressively build up the membrane-embedded factors to reach a minimal machinery for efficient biogenesis of complex membrane proteins thought to exist in the last universal common ancestor^7^. The ability to design membrane proteins of desired characteristics *de novo*^121^ makes this goal feasible. Such a bottom-up reconstruction would define the core design principles and minimal requirements for translocation factors that facilitate membrane protein insertion. These insights might also help understand the mechanisms of other types of insertion factors, such as those in endosymbiont organelle membranes^122–126^ or peroxisomes^127^, that emerged after the evolution of eukaryotes.

## Box 1 Membrane protein topology

Alpha-helical membrane proteins contain one or more transmembrane domains (TMDs). They are typically helices of ~22 predominantly non-polar amino acids, but can be as short as 13 amino acids, as long as 30 amino acids, and can have substantially hydrophilic or even charged character^20,128^. The number, location, and distribution of TMDs is essentially unrestricted. Membrane protein topology can be N_cyt_ or N_exo_ depending on whether the N-terminus is located on the cytosolic or exoplasmic side of the membrane, respectively. Proteins that initiate via their first TMD in the N_cyt_ topology can have a preceding N-terminal cytosolic domain of any length. Proteins that initiate via their first TMD in the N_exo_ topology typically contain a preceding N-terminal translocated domain shorter than ~50 amino acids. Some proteins initiate membrane insertion in the N_cyt_ topology before proteolytic removal of the first hydrophobic domain, leaving the downstream protein in the N_exo_ topology. The processed hydrophobic domain in these cases is termed a cleavable signal peptide, whose characteristically short hydrophobic segment (~7-9 amino acids) allows selective recognition by signal peptidase^27^. N_exo_ membrane proteins produced in this manner typically contain a translocated N-terminal domain longer than ~100 amino acids between the signal peptide and first TMD. The topology of the first hydrophobic domain is determined by a combination of flanking domain charge, flanking domain length, and hydrophobic domain length and hydrophobicity^129^. The topology of downstream TMDs alternate in orientation opposite to the TMD preceding it. The loops between TMDs are typically short (around 15 amino acids on average), but can be of any length regardless of which side of the membrane they reside. The C-terminal hydrophilic domain following the last TMD can be of any length and can be located in the cytosolic or exoplasmic side of the membrane.

## Box 2 Membrane protein translocation machinery

The prokaryotic plasma membrane, eukaryotic ER, and inner membranes of endosymbiont-derived organelles (mitochondrial and plastids) derive from a common ancestor that already had membrane insertion factors of the Oxa1 and SecY families^7,108^, depicted below in blue and green, respectively. The nomenclature for these families across species is heterogeneous (see figure below) due to the manner and order of their discovery^125^. SecY family members in prokaryotes and endosymbiont-derived organelles continue to be called SecY whereas family members in the ER are termed Sec61 (*S. cerevisiae*) or Sec61α. These channel-forming subunits are typically associated with two small membrane proteins which have different names in different organisms (Sec61β and Sec61γ in mammals, SecE and SecG in bacteria). Oxa1 family members in the mitochondrial inner membrane are called Oxa1, those in the inner chloroplast membrane are called Alb3, and those in the bacterial plasma membrane are called YidC. Oxa1 family members in the ER are called GET1, EMC3, and TMCO1, each of which is associated with a partner (GET2, EMC6, and OPTI, respectively) as part of the GET, EMC, and GEL complexes. GET and EMC are widely distributed across most eukaryotes, whereas GEL has a more sporadic distribution. Archaeal Oxa1 most closely resembles the ER counterparts and likely associates with a GET2/EMC6/OPTI-like partner, consistent with an archaeal origin for the ER. A small number of alpha-helical membrane proteins are inserted into the outer membrane of mitochondria and plastids, and possibly the peroxisomal membrane, by more newly evolved and unrelated machinery^122–127^. Beta-barrel membrane proteins, found only in the outer membranes of bacteria, mitochondria and plastids, are inserted by a yet different specialized machinery^130,131^.
